# *In-Silico* Computing of the Most Deleterious nsSNPs in *HBA1* Gene

**DOI:** 10.1371/journal.pone.0147702

**Published:** 2016-01-29

**Authors:** Sayed AbdulAzeez, J. Francis Borgio

**Affiliations:** Institute for Research and Medical Consultation (IRMC), University of Dammam, Dammam, Saudi Arabia; University of Michigan, UNITED STATES

## Abstract

**Background:**

α-Thalassemia (α-thal) is a genetic disorder caused by the substitution of single amino acid or large deletions in the *HBA1* and/or *HBA2* genes.

**Method:**

Using modern bioinformatics tools as a systematic *in-silico* approach to predict the deleterious SNPs in the *HBA1* gene and its significant pathogenic impact on the functions and structure of HBA1 protein was predicted.

**Results and Discussion:**

A total of 389 SNPs in *HBA1* were retrieved from dbSNP database, which includes: 201 non-coding synonymous (nsSNPs), 43 human active SNPs, 16 intronic SNPs, 11 mRNA 3′ UTR SNPs, 9 coding synonymous SNPs, 9 5′ UTR SNPs and other types. Structural homology-based method (PolyPhen) and sequence homology-based tool (SIFT), SNPs&Go, PROVEAN and PANTHER revealed that 2.4% of the nsSNPs are pathogenic.

**Conclusions:**

A total of 5 nsSNPs (G60V, K17M, K17T, L92F and W15R) were predicted to be responsible for the structural and functional modifications of HBA1 protein. It is evident from the deep comprehensive *in-silico* analysis that, two nsSNPs such as G60Vand W15R in *HBA1* are highly deleterious. These “2 pathogenic nsSNPs” can be considered for wet-lab confirmatory analysis.

## Introduction

HBA1 and HBA2 proteins are coded in α-globin genes, such as *HBA1* and *HBA2* respectively. Reduction or lacking in the synthesis of α-globin proteins leads to α-thal, which is commonly caused by deletional defects, but point mutations are also concerned [1–7]. More than 300 mutations were reported till date in HbVar: A database of human hemoglobin variants and thalassemias [8] and in NCBI (National Center for Biotechnology Information). Non-coding synonymous SNPs (nsSNPs) are associated with most of the inherited disorders in humans including α-thal. Detailed *in-silico* analysis on the structural and functional impacts of the variants of *HBA1* gene is scanty. Hence, the study was aimed to reveal the effect of nsSNPs on the synthesis of HBA1 protein, and to distinguish the functionally deleterious nsSNPs using bioinformatics tools. Modern bioinformatics tools were used to identify nsSNPs in *HBA1* gene, which would alter the structure of the protein. Comprehensive *in-silico* studies would identify and scrutinise the most pathogenic mutants of *HBA1* to further confirm their impact on the synthesis of protein in wet lab studies.

## Materials and Methods

### Datasets and SNP retrieval

*HBA1* gene sequence was downloaded during January 2015 from NCBI (Accession: AAK61216.1; [9]. The NCBI-dbSNPs of *HBA1* gene was retrieved by limiting our search only to non-coding, coding synonymous, and 5′ & 3′ un-translated regions in humans. The non-synonymous SNPs were subjected to find their deleterious effects on α-globin protein.

### SIFT Blink for sequence homology

Sorts intolerant from tolerant (SIFT) is a sequence homology-based tool that predicts variation in protein function caused by the change in amino acid sequence [10]. The hypothesis states that the positions that are important for the function of protein should be conserved in protein family, whereas insignificant positions should not be conserved [11]. The native (Accession: AAK61216.1) and mutated α-globin protein’s sequence were submitted as input file to the SIFT server. The SNPs were classified as tolerant (cutoff value of ≥0.05) or deleterious (cutoff value of ≤0.05) based on the prediction score.

### Impact of an amino acid substitution predicted by PolyPhen 2.0

Polymorphism Phenotyping v2 (PolyPhen 2.0) (http://genetics.bwh.harvard.edu/pph2/uses) is an iterative algorithm uses the straight forward comparative and physical considerations to predicts possible impact of substitution of an amino acid on the function and structure of a human protein [12]. The input query was submitted in FASTA format along with the positions of the substitution (native) and substituting amino acids (mutant). The PolyPhen estimates sensitivity, specificity and calculate the PSIC (position-specific independent count) score, for each and every variant. The PolyPhen also estimates the score difference between variants.

### Protein variation effect analyzer (PROVEAN)

The PROVEAN is a web based tool that predicts the changes in the biological functions of a protein due to an amino acid substitution or indel (http://provean.jcvi.org/index.php), based on the sequence clustering and alignment-based scoring. The variants with scores less than -2.5 were considered deleterious [13].

### SNAP2

SNAP2 is a bioinformatic tool to classify the genetic variation based on the neural network, which predicts the changes due to the nsSNPs on the secondary structure and compare the solvent accessibility of the native and mutated protein to distinguish them into effect (+100, strongly predicted) or neutral (-100, strongly predicted) [14]. The FASTA sequence of the native HBA1 protein was provided as input (S1 File). SNAP2 provides a heatmap with the possible substitution at each position of HBA1 protein, where the score >50 is in dark red indicates strong signal for pathogenicity.

### Mutation Cutoff Scanning Matrix (mCSM)

Mutation Cutoff Scanning Matrix (mCSM) predicts the impacts of mutation on the stability of protein through atomic distance patterns surrounding an amino acid residue [15]. The PDB format of HBA1 protein was provided as input with the residual site of the mutation and mutation chain to get the Predicted Stability Change (ΔΔG) in the protein due to a particular mutation. Score <0 for each variant was considered as destabilizing.

#### SNPs&GO

The SNPs&GO algorithm: a support vector machine based web server, was used to predict the impact of variations at HBA1 protein by calculating the functional information such as biological process, cellular component and molecular function, which are arrayed by Gene Ontology (GO) data base [16, 17]. The FASTA sequence of native HBA1 protein and the list of variations were provided as input (S1 File). Probability values >0.5 for each variant was predicted as disease nsSNP.

### Structure modeling and RMSD prediction

Three dimensional structures were designed for the native and the mutated α-globin chains using SWISS MODEL (http://swissmodel.expasy.org/) to evaluate and compare the stability of structure from mutant with native [18]. The 3D structure of native α-globin chain was modeled using automated homology modeling. The 3D structure was generated based on template PDB Id: 1y01.1.B with highest resolution 2.80 Å [19, 20]. The generated structural model was selected and subjected for the structural validation using PROCHECK [21]. The amino acid residue substitutions or mutant structures were generated using the Swiss-Pdb Viewer software [22]. Energy minimization for the native and mutants were done using the GROMACS program [23].

### Trajectory Analysis

#### Identification of stabilizing residues

SRide online server predicts the stabilizing residues (SRs) based on LRO (long range order), stabilization center, surrounding hydrophobicity and conservation score. The differences between native and mutant proteins were compared based on the stabilizing residues [24].

#### Prediction of residue positions

FlexPred, a freely available web-server (http://flexpred.rit.albany.edu/), uses solvent accessibility of a protein sequence to identify the residual positions involved in conformational switches. The conformational switches involved in kinetic energy and causes pathogenic disorders. Server accepts FASTA format and provides conformational changes on each amino acid as rigid (R) or flexible (F).

#### Hydrogen Bond Analysis

Hydrogen Bond Analysis Tool (HBAT) is a program that analysis the changes in hydrogen bonds and its effect on the formations of 3D structure of a protein. It explores the variations between the native and mutants in terms of changes in the hydrogen bonds. This tool analyses the PDB file and provides the angles and distances between hydrogen bonds in macromolecule [25].

#### Molecular dynamic simulation

DelPhi is an online tool, which was used for the molecular dynamic simulation to calculate the total difference in energy at solvated condition of native and mutated proteins. The PDB model structure of both native and mutants were used as input to obtain the grid, coulombic and solvation energies.

## Results and Discussion

### nsSNP retrieval and function prediction

**Fig 1:**
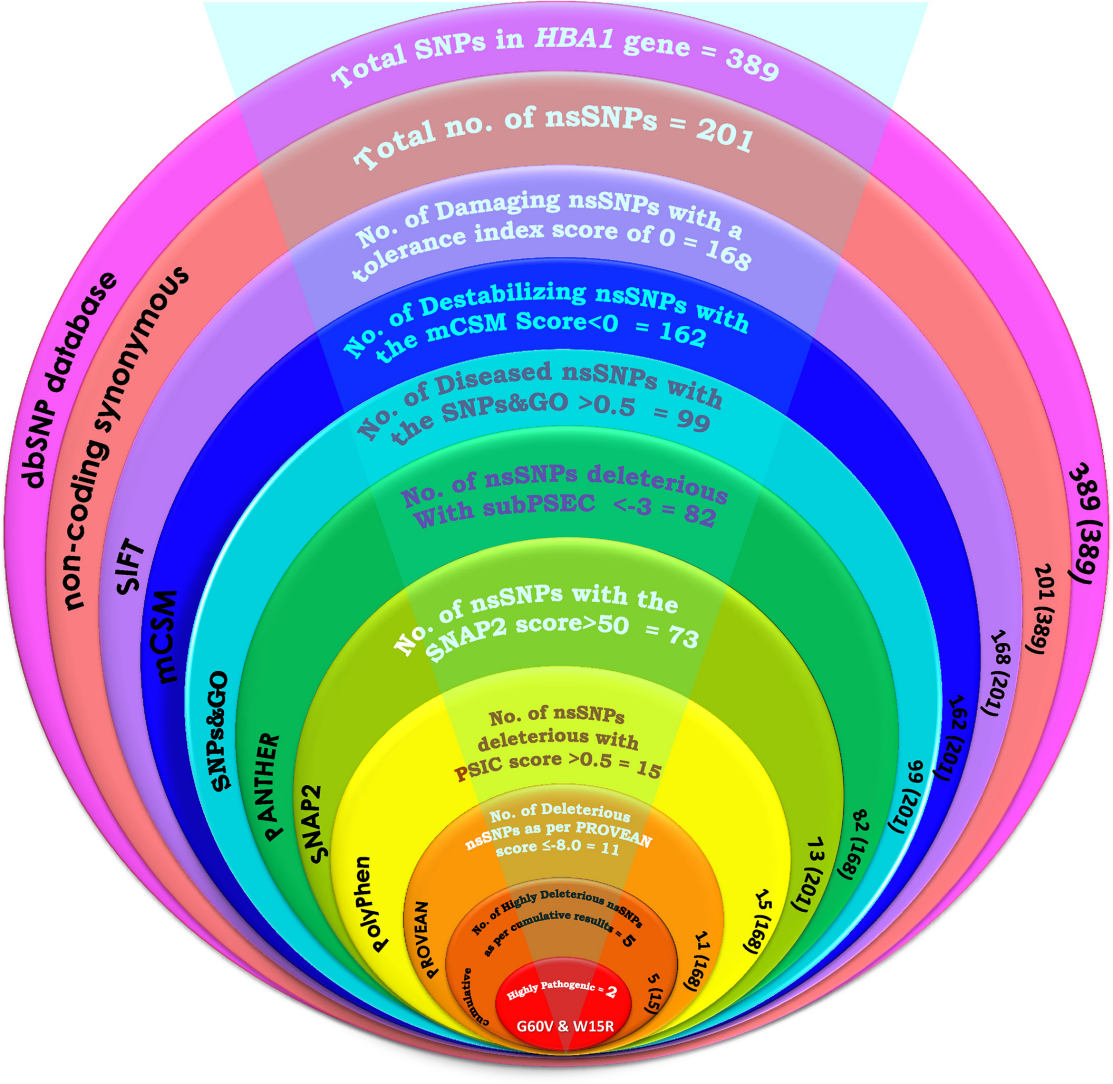
Graphic illustration of bioinformatic tools used for the precise identification of the most deleterious nsSNPs of *HBA1* gene.

**Fig 2:**
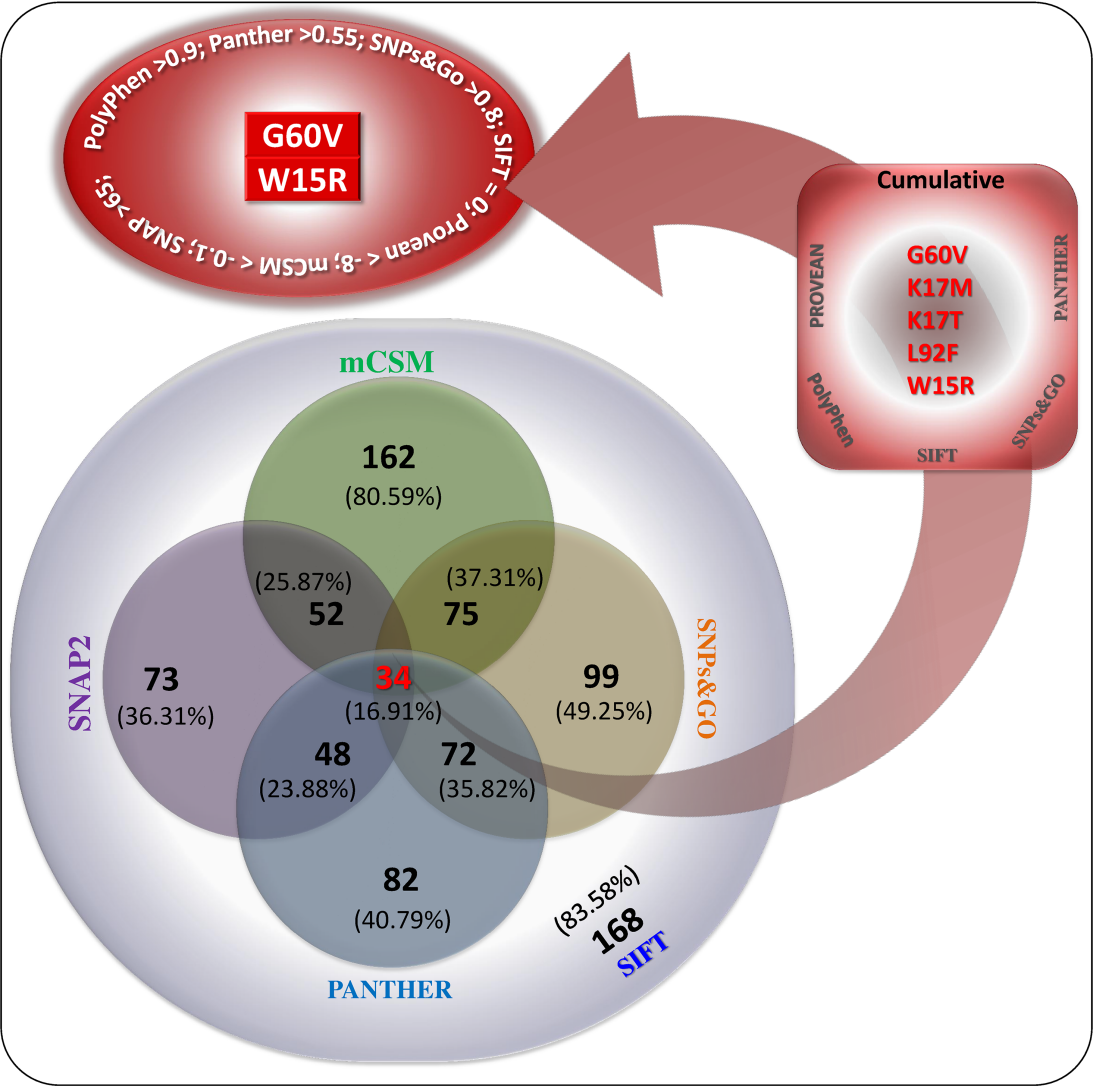
Prediction matching to the highly pathogenic nsSNPs of *HBA1* gene. The 2 highly pathogenic nsSNPs are having the scores: Polyphen >0.9; Panther >0.55; SNPs&Go >0.8; SIFT = 0; PROVEAN < -8; mCSM < -0.1; SNAP2 >65.

A total of 389 SNPs in *HBA1* gene were retrieved from dbSNP database [9]. Which includes: 43 human active SNPs, 201 non-coding synonymous SNPs, 9 coding synonymous SNPs, 11 SNPs in the mRNA 3′ Un-Translated Region (UTR), 9 SNPs in 5′ UTR region, 16 SNPs in intronic regions and the remaining 100 SNPs are of other types. We selected non-coding synonymous SNPs on *HBA1* gene for our investigation and were categorized using the state-of-art-tools such as SIFT, PolyPhen, PROVEAN, SNPs&GO, mCSM, SNAP2 and PANTHER [10, 12, 13, 15, 16, 26]. A total of 168 nsSNP were found to be deleterious with the tolerance index score 0.00 on SIFT platform (Fig 1, S1 Table). Heatmap of the HBA1 protein (S1 Fig) was generated using SNAP2 tool, where, 165 nsSNPs (92 effective nsSNPs: SNAP2 score 0 to 50; 73 highly effective nsSNPs: SNAP2 score 50 to 100) were predicted to be effect and 36 were neutral (SNAP2 score <0 to -100) (Fig 1). A total of 162 nsSNPs were predicted to be destabilizing (mCSM score <0) on the structure of HBA1 protein using the mCSM tool (S2 and S3 Tables). Among the 201 nsSNPs submitted to the PolyPhen server, 15 nsSNPs were predicted to be deleterious for HBA1 protein, based on the PSIC score (>0.5). The deleterious and damaging effects of 82 nsSNPs on HBA1 protein were predicted using the PANTHER (Fig 1). Further the analysis was carried out using the PROVEAN, which predicts the impact of SNP on the biological function of a protein. A total of 11 nsSNPs of *HBA1* gene were predicted to be highly deleterious (score ≤-8) using PROVEAN (Fig 1). Based on the substitution position-specific evolutionary conservation score (subPSEC) using PANTHER, PROVEAN score, SIFT score, SNPs&GO score and prediction matching of highly pathogenic nsSNPs with PSIC score (>0.5) on PolyPhen server, a group of 5 nsSNPs [rs28928878 (G60V), rs35210126 (K17M), rs35210126 (K17T), rs17407508 (L92F) and rs33964317 (W15R)] were predicted to be the most significantly deleterious nsSNPs (Fig 1; Table 1). These 5 nsSNPS were cumulatively considered as highly deleterious as they were agreed 100% by PANTHER, PolyPhen, Provean, SIFT and SNPs&GO as deleterious (Table 1, Fig 2, S1 and S4 Tables). While the mCSM disagree the result of the K17M by other tools (Table 1). Even though the SNAP2 agreed K17M, K17T and L92F as effect, the score is <50 (Table 1). The effect of nsSNPs on the sequence conservation, structural impute and sequence attributes were considered for the selection of the highly pathogenic variants [27].

**Table 1:** Cumulative prediction of possible deleterious nsSNPs.

**S. No**	**SNP**^**✝**^	Amino acid change	Ployphen	PANTHER SubPSEC	PROVEAN score	Prediction (Cutoff = -2.5)	mCSM score (ΔΔG)	SNAP2 Score	SNPs&GO
1	rs28928878	G60V*	0.969	-3.68979	-8.355	Deleterious	-0.163	67	0.815
2	rs35210126	K17M	0.615	-4.33503	-5.606	Deleterious	0.16	17	0.718
3	rs35210126	K17T	0.615	-3.36061	-5.575	Deleterious	-0.308	28	0.824
4	rs17407508	L92F	0.997	-5.74786	-3.47	Deleterious	-0.912	41	0.742
5	rs33964317	W15R*	0.985	-3.32531	-12.664	Deleterious	-2.276	77	0.853

Protein ID: NP_000508

^✝^The listed 5 nsSNPs are predicted as DAMAGING or deleterious or effect and agreed by PolyPhen, Panther, SNPs&Go, Provean.

* The highly pathogenic nsSNPs were agreed unanimously by all the tools with the scores: PolyPhen >0.9; Panther >0.55; SNPs&Go >0.8; SIFT = 0; Provean < -8; mCSM < -0.1; SNAP2 >65.

During the prediction matching analysis, the nsSNPs rs33964317 (W15R) and rs28928878 (G60V) were agreed by the state-of-the-art tools, PolyPhen (>0.9), PANTHER (>0.55), SNPs&Go (>0.8), SIFT (= 0), Provean (< -8), mCSM (< -0.1) and SNAP2 (>65) as highly deleterious nsSNPs on *HBA1* gene (Fig 2, Table 1). All the tools, PolyPhen, PANTHER, Provean, SNPs&GO and SNAP2 were unanimously agreed the highly deleterious nature of G60V and W15R (Fig 2, Table 1). Analysis of 201 nsSNPs of *HBA1* gene for the prediction of pathogenic nsSNPs were almost similar (82.0%) for the SIFT and mCSM. More than 80% of overlapped similarity was observed between the SIFT and mCSM on pathogenic nsSNPs (Fig 2). Almost 50% of the predictions of pathogenic nsSNPs were found to be disagreed between SIFT and SNPs&GO. About 16.91% of the nsSNPs were agreed as deleterious by the SIFT, SNAP2, SNPs&GO, PANTHER and mCSM. The percentage of disagree of the pathogenicity of *HBA1*-nsSNPs between different tools were comparatively lesser than the previous studies on different set of nsSNPs [14]. The selected state-of-the-art tools have covered maximum number of methods (AS alignment score; NN neural networks; HMM hidden Markov models; SVM support vector machine; BC Bayesian classification) used for the prediction of pathogenic nsSNPs [27]. The prediction from SNAP2, mCSM, PROVEAN, SNPs&Go, PANTHER, SIFT and PloyPhen were found to be significant (*p = 4*.*5227E-255* of single factor ANOVA test) and some of the predictions were highly correlated (S2 Fig). Student T-test between the tools were significant at *p <0*.*0001*. It is evident that, the selected tools are sufficient to predict the pathogenicity of the nsSNPs.

### Structure Modeling and Stability Check

**Fig 3:**
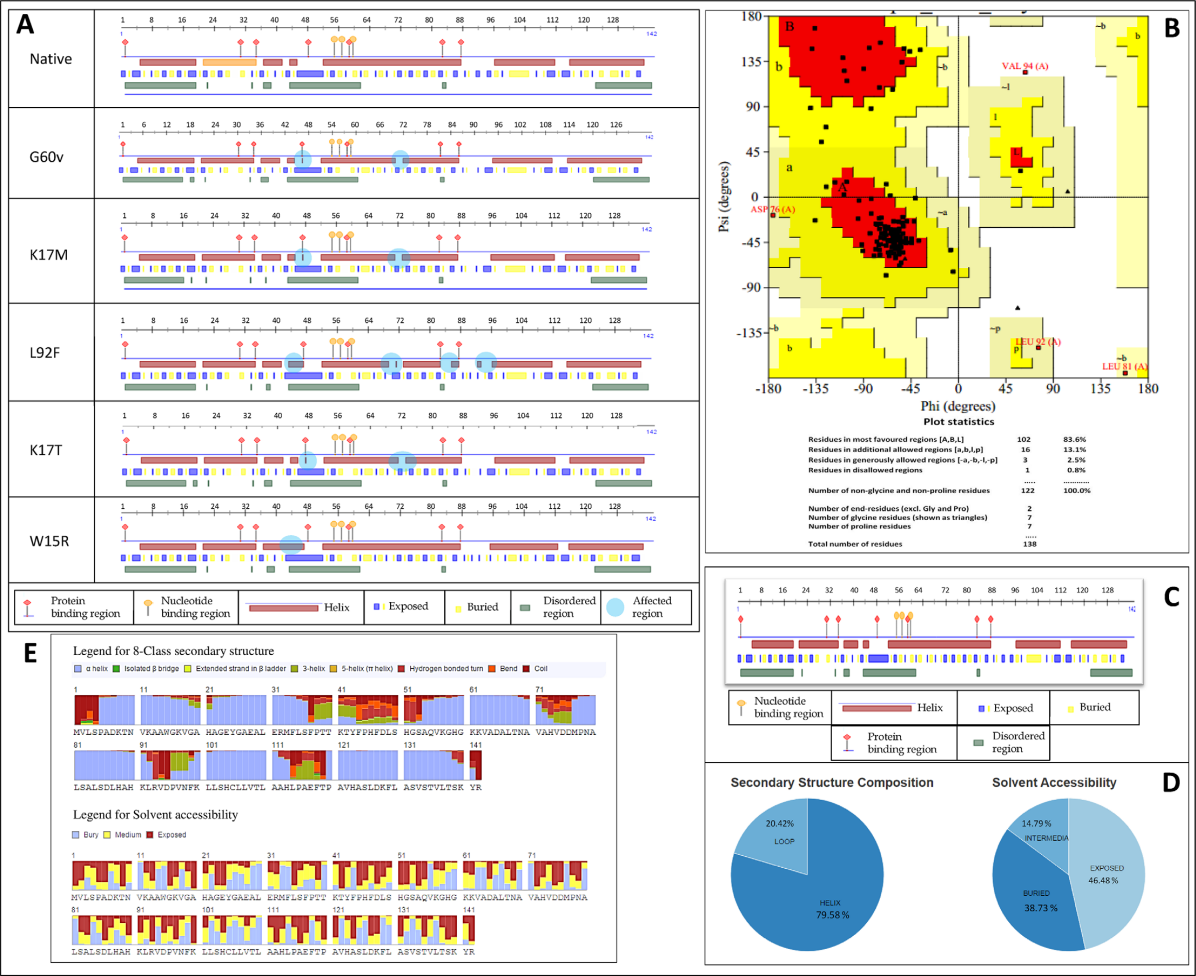
A: Comparing the secondary structure of the mutated and native HBA1 protein. B: Ramachandran plot of constructed HBA1 protein. Most of the amino acid residues were in the most favored region. C: Protein binding regions in the secondary structure of HBA1 protein. D: States of the secondary structure. E: Eight class Secondary structure of HBA1 protein by RaptorX.

The 3D structure for HBA1 protein was modeled based on a template PDB id-1y01.1.B (From ExPDB) to compare and understand the significant effect of mutations, in the stability of protein structures and structure–function relationships [28,29]. The template's quality of 1y01.1.B was found to be the highest, and was used to build a model using Promod-II program [22, 30]. 1y01.1.B was recognized as hemoglobin subunit alpha chain, which involved in oxygen transport, from the lung to the various peripheral tissues. These observations have confirmed that the modeled template resembles HBA1 protein. The QMEAN6 (0–1), reliability and Z- scores (0–1) were calculated for the whole protein model [31]. The modeled structure was validated using PROCHECK [21]. The secondary structure was subjected for the analysis of Ramachandran plot. The resulted structure obeyed all the restrictions based on potential energy calculations. A total of 83.6% (102 out of 138) of core residues of HBA1 protein were in the most favored region in Ramachandran plot, and only few amino acids were deviated (Fig 3B) [32].

### Trajectory Analysis

Two (MET33 and GLY60) stabilizing residues were identified in HBA1 protein through SRide server [24, 33] (Table 2. One of the stabilizing residues is missing in the G60V and L92F, this could influence the structure of the protein. The RMSD (Å) values are significantly deviated from the native. These results were co-inside with the cumulative results obtained based on PolyPhen, PROVEAN, SNPs&GO, SIFT and PANTHER (Table 1). The total energy (kJ/mol) values of mutated protein are also significantly deviated from the native protein, which could influence the structure and biological functions of the mutated HBA1 proteins (Table 2). Reduced RMSD (Å) and increased total energy (kJ/mol) (G60V) OR vice versa (W15R) have been observed from the highly pathogenic candidate nsSNPs. The divergence in the RMSD and total energy (kJ/mol) in the 5 deleterious mutated proteins is mainly due to the substitutions, which could affect the functional activity and the stability of the mutated protein [34–36]. Analysis using INTERPRED confirmed that the mutated HBA1 was a non-repair protein with the predicted values of -0.056, 7.00e-001 and -0.691. Three states of secondary structure were predicted in HBA1 protein: helix (H; includes alpha-, pi- and 3_10-helix), β strand (E = extended strand in beta-sheet conformation of at least two residues length) and loop (L) (Fig 3C & 3D). Secondary structure of *HBA1* was predicted with an expected average accuracy of >72% by a system of neural networks [37]. DelPhi results for the native and highly deleterious mutants were diversified in grid energies, coulombic, and solvation energies (Table 3).

**Table 2:** Total energy and RMSD of deleterious nsSNPs.

S. no.	SNP	Residue change	RMSD (Å)	Total energy (kJ/mol) Mutant	SRide Stabilizing residues
1	Native vs template*	NA	0.41	3290	NA
2	Native	NA	0	3356	MET33, GLY60
3	rs28928878	G60V	0.03	3970	MET33
4	rs35210126	K17M	3.94	3709	MET33, GLY60
5	rs35210126	K17T	4.42	3710	MET33, GLY60
6	rs17407508	L92F	0.14	3385	MET33
7	rs33964317	W15R	1.5	3107	MET33, GLY60

* Designed Native Model vs the PDB temple 1y01.1.B. NA: Not applicable

**Table 3:** Molecular dynamic simulation of native and mutant HBA1.

Substitution	Total grid energy (KT)	Solvation energy (KT)	Coulombic energy (KT)
Native	45399.8	40485	22646.7
G60V	47499.6	39878	36368.8
K17M	47101.0	39665	34253.0
K17T	44308.5	40095	31206.6
L92F	45541.0	39550	35261.2
W15R	44525.2	38875	30282.0

The FlexPred was used for solvent accessibility to foresee the residual positions, which are involved in the conformational switches of HBA1 protein. It was identified that the rs17407508 in L92F mutant positions at 7, 53 and 60 amino acid residues, which were flexible and involved in variety of biological functions including pathogenic disorders. The RaptorX server was applied to check the secondary structure and solvent accessibility of HBA1 protein. The percentage of disorder predicted by RaptorX server was between 46 and 59 amino acids (Fig 3E). Eight classes of secondary structure were predicted in HBA1 protein using RaptorX server which were α helix, isolated β bridge, 3-helix, 5-helix (π helix), extended strand in β ladder, hydrogen bonded turn, coil and bend [38].

To reconfirm the secondary structure and solvent accessibility, we used the predict protein server [39]. The composition of secondary structure of HBA1 protein was divided as protein binding region, helix, buried and disordered region. Three states of secondary structure were predicted in HBA1 protein: helix 79.58% (H; includes α-, pi- and 3_10-helix), β-strand (E = extended strand in β-sheet conformation of at least two residues length) and loop (L) 20.42% (Fig 3C & 3D). Results of Solvent accessibility of the secondary structure of HBA1 protein were 14.79% intermedia, 38.73% buried residues and 46.48% exposed residues (Fig 3C & 3D).

**Fig 4:**
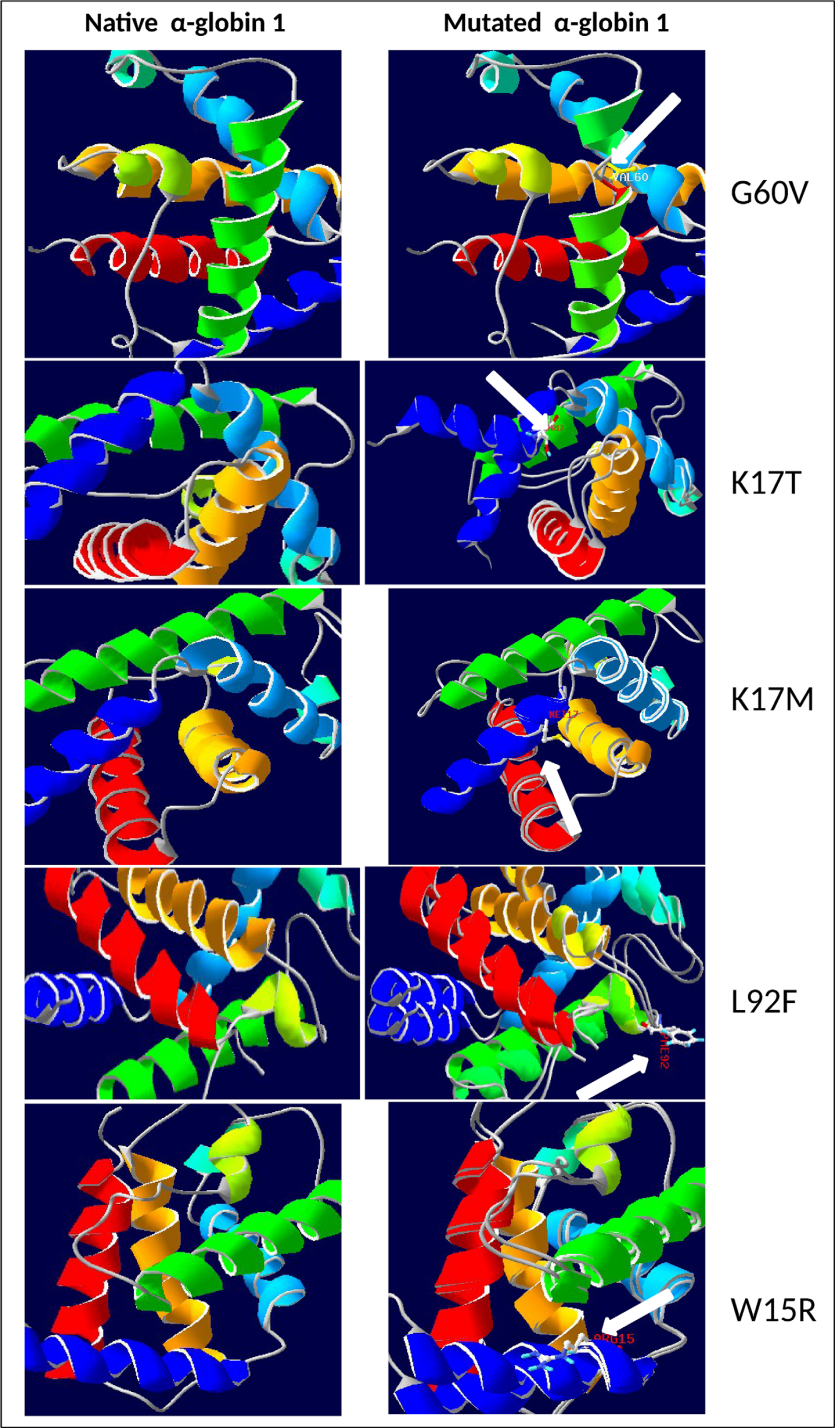
Super imposed 3D structures of the native and highly deleterious mutated HBA1 proteins.

The structural annotations were done by online based predictprotein.org server [40] and NORSp (NOn-Regular Secondary Structure), which identified 7 disordered regions in HBA1 protein. Compare to the native structure of HBA1 protein, mutant G60V has 9 helixes with methionine and valine at 1 and 2; arginine at 32 position, serine at 36 position; leucine and serine at 49 and 50 position; lysine at 61 and 62 position; aspartic acid at 86 position; and lysine at 91 position. Nine and 6 helixes were formed due to L92F, and W15R respectively. K17M has 20 residues in disordered region out of 45 to 64 amino acid residues (Table 4) and W15R protein had one helix less compared to the native protein (Fig 3A; Table 4). Three dimension structure were drawn for the native and mutants proteins using SWISS-PDB, which explicitly shows the structural alterations upon mutations (Fig 4). The changes predicted on the sequence based homology modeling between native and the five pathogenic substitutions on the stability of the HBA1 protein, supports the pathogenicity of the five deleterious substitutions. The highly pathogenic substitutions were agreed unanimously by all the tools with the scores PolyPhen >0.9; Panther >0.55; SNPs&Go >0.8; SIFT = 0; Provean < -8; mCSM < -0.1; SNAP2 >65. This observation clearly indicates that the threshold score for the state-of-the-art tools can be narrowed to predict the highly pathogenic substitutions. Combination of the analysis from various state-of-the-art tools to be the best method, instead to follow a single tool to prioritize the pathogenic nsSNP.

**Table 4:** Comparing the helix, protein binding, Disordered region and exposed/buried residues.

nsSNP	Protein binding region	Helix	Exposed	Buried	Disordered region
native	7 Protein binding region and 3 polynucleotide binding region	7	34	32	7
G60V	7 Protein binding region and 3 polynucleotide binding region	9	34	35	7
K17M	7 Protein binding region and 3 polynucleotide binding region	9	33	33	8
L92F	7 Protein binding region and 3 polynucleotide binding region	11	33	33	7
K17T	7 Protein binding region and 3 polynucleotide binding region	9	33	35	8
W15R	7 Protein binding region and 3 polynucleotide binding region	6	34	36	7

## Conclusion

Analysis using various state-of-the-art tools predicted the influence of nsSNPs on the functional and structural deviations in HBA1 protein. Structural homology-based method and sequence homology-based tools on the HBA1 protein have scrutinised 5 nsSNPs as damaging SNPs [rs28928878 (G60V), rs35210126 (K17M), rs35210126 (K17T), rs17407508 and rs33964317]. The stepwise prediction of pathogenicity of nsSNPs [SIFT > mCSM > SNPs&Go > PANTHER > SNAP2 > PolyPhen > Provean > Cumulative], prediction matching among the tools and the trajectory analysis revealed that the rs33964317 (W15R) and rs28928878 (G60V) were the most damaging and highly deleterious nsSNPs affecting the stability of the HBA1 protein. These two highly pathogenic substitutions can be considered for the detailed wet lab confirmatory analysis.

## Supporting Information

S1 Fig Heatmap of HBA1 protein generated by using SNAP2. (TIFF)

S2 Fig Surface chart of correlation between the predication by various tools. (TIFF)

S1 File List of the FASTA sequences of HBA1 protein used in the study. (DOCX)

S1 Table SIFT score and the predicted effect of the variants on HBA1 protein. (DOCX)

S2 Table SNAP2 score and the predicted effect of the variants on HBA1 protein. (DOCX)

S3 Table Wild and mutant residues of HBA1 protein and mCSM score. (DOCX)

S4 Table Prediction and probability of variants of HBA1 protein using SNPs&GO. (DOCX)
