# Supplementary figures and images for: In-Silico Computing of the Most Deleterious nsSNPs in HBA1 Gene
Source: PLoS One. 2016 Jan 29;11(1):e0147702. doi: 10.1371/journal.pone.0147702 (PMC4733110; doi:10.1371/journal.pone.0147702)

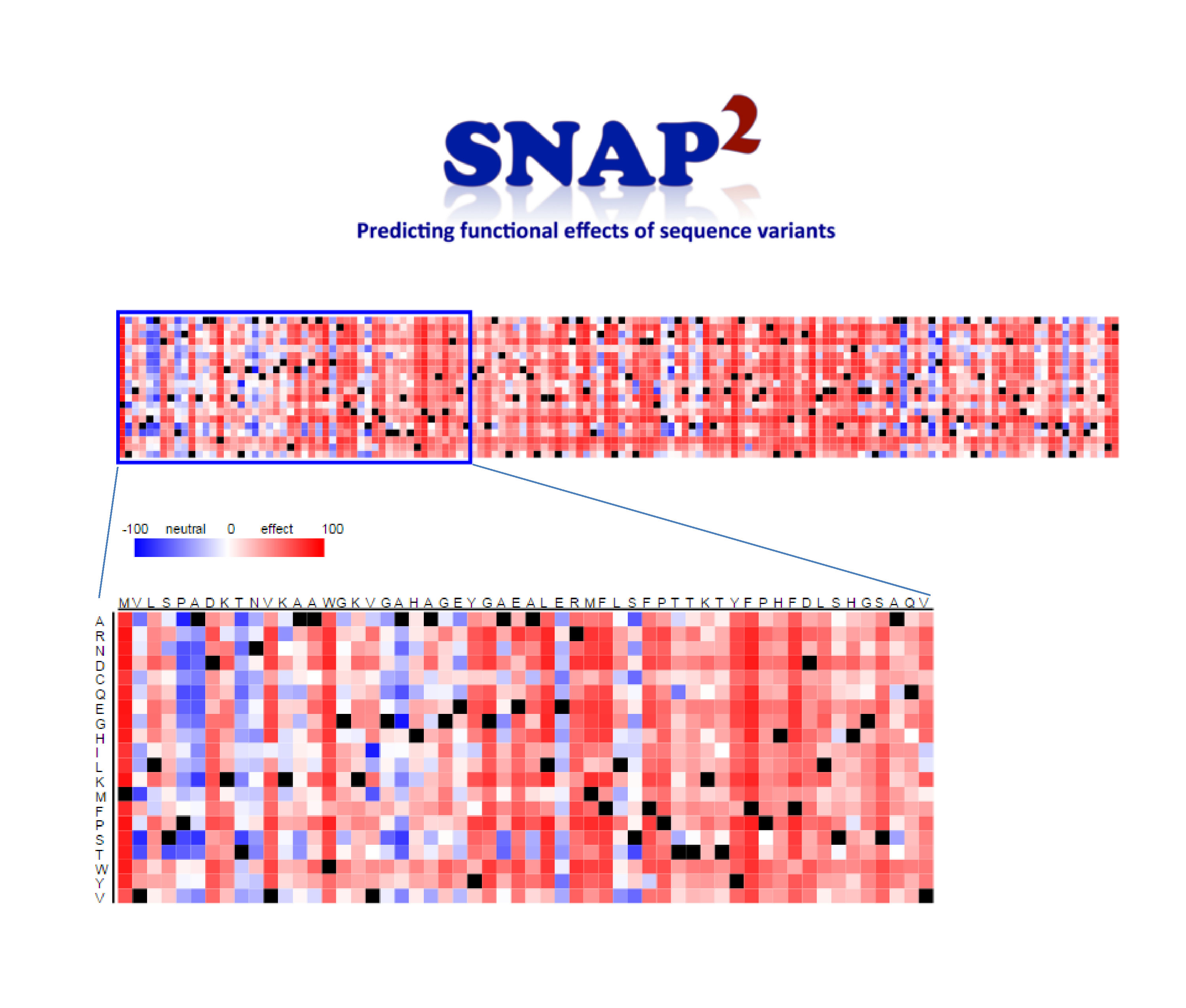

Supplement: S1 Fig — (TIFF) [file pone.0147702.s001.tiff]

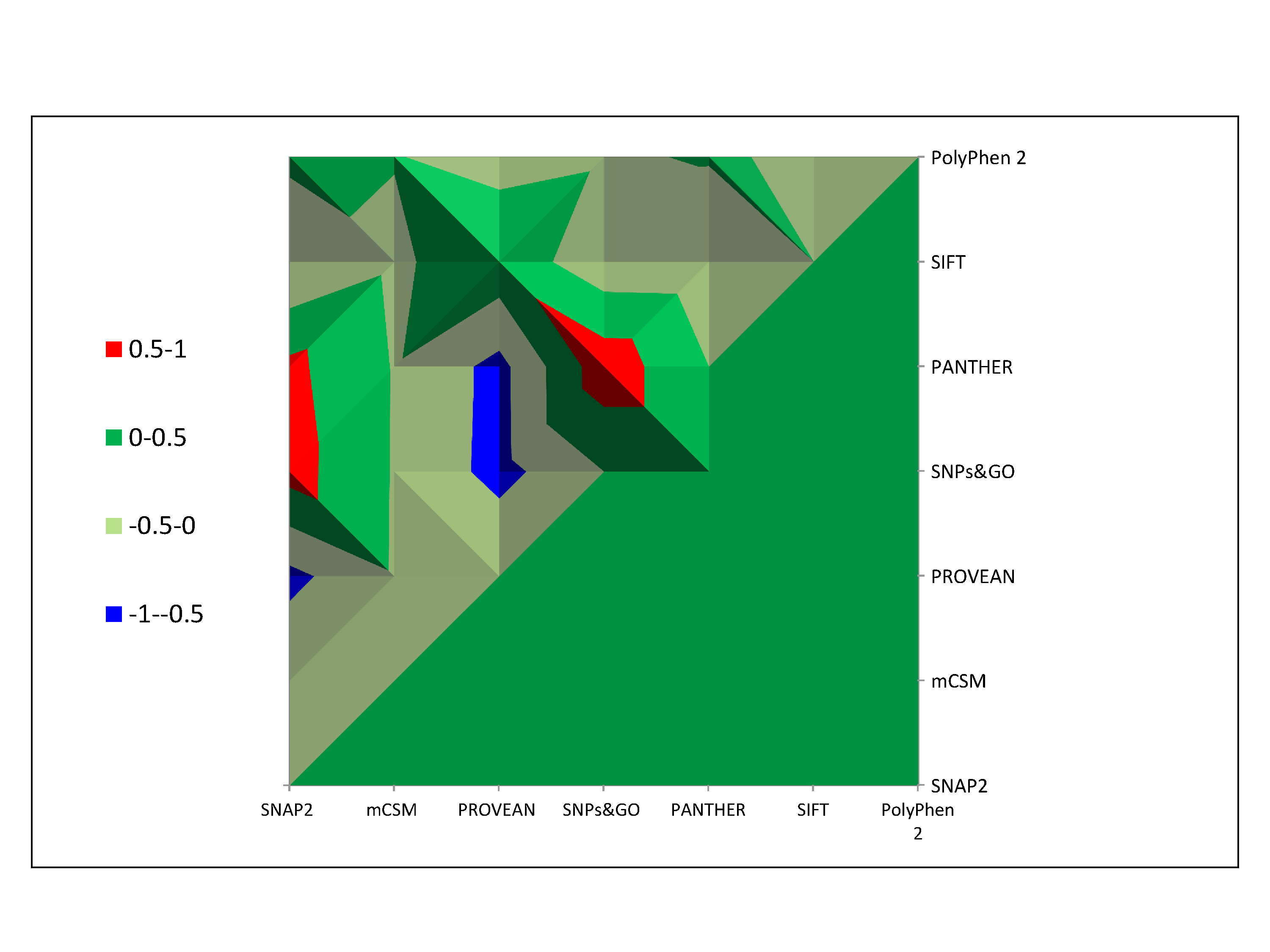

Supplement: S2 Fig — (TIFF) [file pone.0147702.s002.tiff]
