# Supplementary material for: In-Silico Computing of the Most Deleterious nsSNPs in HBA1 Gene
Source: PLoS One. 2016 Jan 29;11(1):e0147702. doi: 10.1371/journal.pone.0147702 (PMC4733110; doi:10.1371/journal.pone.0147702)
Supplement: S1 File — (DOCX) [file pone.0147702.s003.docx]

**S1 File.** List of the FASTA sequences of HBA1 protein used in the study.

>Native

MVLSPADKTNVKAAWG**K**VGAHAGEYGAEALERMFLSFPTTKTYFPHFDLSHGSAQVKGHGKKVADALTNA

VAHVDDMPNALSALSDLHAHK**L**RVDPVNFKLLSHCLLVTLAAHLPAEFTPAVHASLDKFLASVSTVLTSK

YR

>G60V

MVLSPADKTNVKAAWGKVGAHAGEYGAEALERMFLSFPTTKTYFPHFDLSHGSAQVKGH**V**KKVADALTNA

VAHVDDMPNALSALSDLHAHKLRVDPVNFKLLSHCLLVTLAAHLPAEFTPAVHASLDKFLASVSTVLTSK

YR

>K17M

MVLSPADKTNVKAAWG**M**VGAHAGEYGAEALERMFLSFPTTKTYFPHFDLSHGSAQVKGHGKKVADALTNA

VAHVDDMPNALSALSDLHAHKLRVDPVNFKLLSHCLLVTLAAHLPAEFTPAVHASLDKFLASVSTVLTSK

YR

>K17T

MVLSPADKTNVKAAWG**T**VGAHAGEYGAEALERMFLSFPTTKTYFPHFDLSHGSAQVKGHGKKVADALTNA

VAHVDDMPNALSALSDLHAHKLRVDPVNFKLLSHCLLVTLAAHLPAEFTPAVHASLDKFLASVSTVLTSK

YR

>L92F

MVLSPADKTNVKAAWGKVGAHAGEYGAEALERMFLSFPTTKTYFPHFDLSHGSAQVKGHGKKVADALTNA

VAHVDDMPNALSALSDLHAHK**F**RVDPVNFKLLSHCLLVTLAAHLPAEFTPAVHASLDKFLASVSTVLTSK

YR

>W15

MVLSPADKTNVKAA**R**GKVGAHAGEYGAEALERMFLSFPTTKTYFPHFDLSHGSAQVKGHGKKVADALTNA

VAHVDDMPNALSALSDLHAHKLRVDPVNFKLLSHCLLVTLAAHLPAEFTPAVHASLDKFLASVSTVLTSK

YR
