# Supplementary material for: In-Silico Computing of the Most Deleterious nsSNPs in HBA1 Gene
Source: PLoS One. 2016 Jan 29;11(1):e0147702. doi: 10.1371/journal.pone.0147702 (PMC4733110; doi:10.1371/journal.pone.0147702)
Supplement: S1 Table — (DOCX) [file pone.0147702.s004.docx]

**S1 Table.** SIFT score and the predicted effect of the variants on HBA1 protein.

| **SNP** | **Allele** | **Amino acid change** | **Protein ID** | **Amino acid** | **SIFT Prediction (Orthologs)** | **Score (Orthologs)** |
| --- | --- | --- | --- | --- | --- | --- |
| rs63749948 | A/C/T | A111D | NP_000549 | D | DAMAGING | 0 |
| rs34629158 | A/G | A111T | NP_000508 | T | DAMAGING | 0 |
| rs34863047 | A/G | A112T | NP_000508 | T | TOLERATED | 0.21 |
| rs34204059 | A/C/T | A116D | NP_000508 | D | TOLERATED | 0.1 |
| rs63749927 | A/C | A121E | NP_000549 | E | DAMAGING | 0 |
| rs28928881 | A/C/G/T | A124S | NP_000549 | S | DAMAGING | 0 |
| rs63750613 | A/C/T | A131V | NP_000549 | V | DAMAGING | 0 |
| rs35615982 | A/C | A13D | NP_000508 | D | DAMAGING | 0.05 |
| rs35331909 | A/C/G | A14P | NP_000508 | P | DAMAGING | 0 |
| rs35628685 | A/C | A20E | NP_000508 | A | TOLERATED | 0.21 |
| rs11548605 | A/C/G | A22D | NP_000508 | D | DAMAGING | 0 |
| rs34324664 | C/G | A22P | NP_000508 | P | DAMAGING | 0 |
| rs11548605 | A/C/G | A22V | NP_000508 | V | TOLERATED | 0.05 |
|  |  | A27V |  |  |  |  |
| rs34574239 | A/C | A54V | NP_000508 | V | DAMAGING | 0 |
| rs34502246 | A/C | A64D | NP_000508 | D | DAMAGING | 0 |
| rs34733452 | C/T | A66V | NP_000508 | V | DAMAGING | 0 |
| rs34090856 | A/C | A6D | NP_000508 | D | TOLERATED | 0.1 |
| rs34751764 | C/G | A6P | NP_000508 | P | DAMAGING | 0.01 |
|  |  | A72E |  |  |  |  |
|  |  | A72G |  |  |  |  |
| rs3180281 | A/C/G/T | A72V | NP_000508 | V | DAMAGING | 0 |
| rs34586189 | A/G | A80T | NP_000508 | T | DAMAGING | 0.03 |
| rs34879587 | A/C | A83D | NP_000508 | D | TOLERATED | 0.1 |
| rs63750676 | A/G | A83T | NP_000508 | T | TOLERATED | 0.08 |
| rs35239527 | G/T | A89S | NP_000508 | S | DAMAGING | 0 |
| rs35059618 | A/T | C105S | NP_000549 | S | TOLERATED | 0.07 |
| rs63751204 | C/G | D127E | NP_000549 | E | DAMAGING | 0 |
| rs63750467 | A/C/G/T | D127G | NP_000549 | G | DAMAGING | 0 |
| rs63750950 | A/C/G/T | D127H | NP_000549 | H | DAMAGING | 0 |
| rs63750950 | A/C/G/T | D127N | NP_000549 | N | DAMAGING | 0 |
| rs63750467 | A/C/G/T | D127V | NP_000549 | V | DAMAGING | 0 |
| rs63750950 | A/C/G/T | D127Y | NP_000549 | Y | DAMAGING | 0 |
| rs33944368 | A/C/G | D48A | NP_000508 | A | DAMAGING | 0 |
| rs33944368 | A/C/G | D48G | NP_000508 | G | DAMAGING | 0 |
| rs34269448 | A/C/G/T | D48H | NP_000508 | H | DAMAGING | 0 |
| rs35873730 | A/G | D65G | NP_000508 | G | DAMAGING | 0 |
| rs33984024 | A/C/G/T | D65H | NP_000508 | H | DAMAGING | 0 |
| rs33984024 | A/C/G/T | D65N | NP_000508 | N | DAMAGING | 0.02 |
| rs33984024 | A/C/G/T | D65Y | NP_000508 | Y | DAMAGING | 0 |
| rs33921047 | A/C/G/T | D75A | NP_000508 | A | DAMAGING | 0 |
|  |  | D75G |  |  |  |  |
| rs28928875 | A/C/G | D75H | NP_000508 | H | DAMAGING | 0 |
| rs28928875 | A/C/G | D75N | NP_000508 | N | DAMAGING | 0 |
| rs33977363 | A/C/G/T | D76H | NP_000508 | H | DAMAGING | 0 |
| rs33977363 | A/C/G/T | D76N | NP_000508 | N | TOLERATED | 0.05 |
| rs33991223 | A/C/G | D76V | NP_000508 | V | DAMAGING | 0 |
| rs33977363 | A/C/G/T | D76Y | NP_000508 | Y | DAMAGING | 0 |
| rs33986902 | A/C/G/T | D7A | NP_000508 | A | DAMAGING | 0 |
| rs33986902 | A/C/G/T | D7G | NP_000508 | G | DAMAGING | 0 |
| rs33961916 | A/C/G/T | D7N | NP_000508 | N | DAMAGING | 0 |
| rs33986902 | A/C/G/T | D7V | NP_000508 | V | DAMAGING | 0 |
| rs33961916 | A/C/G/T | D7Y | NP_000508 | Y | DAMAGING | 0 |
| rs33915947 | A/C/G/T | D86N | NP_000508 | N | DAMAGING | 0 |
| rs33915947 | A/C/G/T | D86Y | NP_000508 | Y | DAMAGING | 0 |
| rs34814612 | C/G | D95E | NP_000508 | E | DAMAGING | 0 |
| rs28928879 | A/C/G | D95G | NP_000549 | G | DAMAGING | 0 |
| rs34102339 | A/C/G/T | D95H | NP_000508 | H | DAMAGING | 0 |
| rs34102339 | A/C/G/T | D95N | NP_000508 | N | DAMAGING | 0 |
| rs34102339 | A/C/G/T | D95Y | NP_000508 | Y | DAMAGING | 0 |
| rs35932809 | A/C | E117A | NP_000508 | A | DAMAGING | 0.01 |
| rs63749882 | A/C/G | E117K | NP_000549 | K | DAMAGING | 0.01 |
| rs63749882 | A/C/G | E117Q | NP_000549 | Q | DAMAGING | 0.01 |
| rs33939421 | A/G/T | E24G | NP_000508 | G | DAMAGING | 0 |
| rs33939421 | A/G/T | E24V | NP_000508 | V | DAMAGING | 0 |
| rs41530750 | A/C/G/T | E28D | NP_000508 | D | DAMAGING | 0.02 |
| rs34776279 | A/G | E28K | NP_000508 | K | DAMAGING | 0 |
| rs33964507 | A/C/G/T | E28V | NP_000508 | V | DAMAGING | 0 |
| rs33946121 | A/C/T | E31A | NP_000508 | A | DAMAGING | 0 |
| rs33993166 | A/C/G | E31Q | NP_000508 | Q | DAMAGING | 0 |
| rs33946121 | A/C/T | E31V | NP_000508 | V | DAMAGING | 0 |
| rs35511459 | G/T | F44V | NP_000508 | V | DAMAGING | 0 |
| rs35816645 | C/G/T | G16R | NP_000508 | R | DAMAGING | 0.02 |
| rs35993097 | A/G | G19D | NP_000508 | D | DAMAGING | 0 |
| rs34504387 | C/G/T | G19R | NP_000508 | R | DAMAGING | 0 |
| rs34608326 | A/G | G23D | NP_000508 | D | DAMAGING | 0 |
| rs35934411 | A/G | G52D | NP_000508 | D | DAMAGING | 0 |
| rs33960522 | A/C/G | G52R | NP_000508 | R | DAMAGING | 0 |
| rs33960522 | A/C/G | G52S | NP_000508 | S | DAMAGING | 0 |
| rs36062788 | A/G | G58D | NP_000508 | D | DAMAGING | 0 |
| rs35252931 | C/G | G58R | NP_000508 | R | DAMAGING | 0 |
| rs28928878 | A/G/T | G60A | NP_000508 | A | DAMAGING | 0 |
| rs28928878 | A/G/T | G60D | NP_000508 | D | DAMAGING | 0 |
| rs28928878 | A/G/T | G60V | NP_000508 | V | DAMAGING | 0 |
| rs35329201 | A/G | H104R | NP_000549 | R | DAMAGING | 0 |
| rs28928884 | C/T | H104Y | NP_000549 | Y | DAMAGING | 0 |
| rs34830032 | C/G | H113D | NP_000508 | D | DAMAGING | 0 |
| rs34713708 | A/G | H113R | NP_000508 | R | DAMAGING | 0 |
| rs63750922 | C/T | H123Y | NP_000549 | Y | DAMAGING | 0 |
| rs34708054 | C/G/T | H21D | NP_000508 | D | DAMAGING | 0.01 |
| rs33943087 | A/C/G | H21P | NP_000508 | P | DAMAGING | 0 |
| rs33943087 | A/C/G | H21R | NP_000508 | R | DAMAGING | 0 |
| rs33931984 | C/G/T | H46D | NP_000508 | D | DAMAGING | 0 |
| rs28928883 | A/C/G | H46P | NP_000508 | P | DAMAGING | 0 |
| rs28928883 | A/C/G | H46R | NP_000508 | R | DAMAGING | 0 |
| rs33931984 | C/G/T | H46Y | NP_000508 | Y | DAMAGING | 0 |
| rs33967561 | A/G/T | H51L | NP_000508 | L | DAMAGING | 0 |
| rs33966883 | A/C/G | H51Q | NP_000508 | Q | DAMAGING | 0 |
| rs33967561 | A/G/T | H51R | NP_000508 | R | DAMAGING | 0 |
| rs35213748 | C/T | H59Y | NP_000508 | Y | DAMAGING | 0 |
| rs36104787 | C/G/T | H73D | NP_000508 | D | DAMAGING | 0 |
| rs35859529 | A/G | H73R | NP_000508 | R | DAMAGING | 0 |
| rs33976776 | A/C/G | H88P | NP_000508 | P | DAMAGING | 0 |
| rs33976776 | A/C/G | H88R | NP_000508 | R | DAMAGING | 0 |
| rs28928876 | A/C/G/T | H88Y | NP_000508 | Y | DAMAGING | 0 |
| rs33944813 | A/C/G/T | H90L | NP_000508 | L | DAMAGING | 0 |
| rs33944813 | A/C/G/T | H90P | NP_000508 | P | DAMAGING | 0 |
| rs33944813 | A/C/G/T | H90R | NP_000508 | R | DAMAGING | 0.01 |
| rs34806456 | A/G | K100E | NP_000508 | E | DAMAGING | 0 |
| rs34273731 | G/T | K100N | NP_000508 | N | DAMAGING | 0 |
| rs63749865 | C/G/T | K128N | NP_000549 | N | DAMAGING | 0 |
| rs63751308 | A/C | K128T | NP_000549 | T | DAMAGING | 0 |
| rs33938574 | A/C/G | K12E | NP_000508 | E | DAMAGING | 0 |
| rs33938574 | A/C/G | K12Q | NP_000508 | Q | DAMAGING | 0 |
| rs33973086 | A/G | K140E | NP_000549 | E | DAMAGING | 0 |
| rs34849179 | A/C | K140T | NP_000549 | T | DAMAGING | 0 |
| rs41407250 | A/G | K17E | NP_000508 | E | DAMAGING | 0 |
| rs35210126 | A/C/T | K17M | NP_000508 | M | DAMAGING | 0 |
| rs35210126 | A/C/T | K17T | NP_000508 | T | DAMAGING | 0 |
| rs34492931 | A/G | K41E | NP_000508 | E | DAMAGING | 0 |
| rs41416747 | A/C/T | K41M | NP_000508 | M | DAMAGING | 0 |
| rs28928886 | C/G | K41N | NP_000508 | N | DAMAGING | 0 |
| rs34182019 | A/G | K57E | NP_000508 | E | DAMAGING | 0 |
| rs33949106 | A/C/G | K57R | NP_000508 | R | DAMAGING | 0.01 |
| rs33949106 | A/C/G | K57T | NP_000508 | T | DAMAGING | 0 |
| rs34259907 | A/G | K61E | NP_000508 | E | DAMAGING | 0.04 |
| rs41381645 | A/C | K62T | NP_000508 | T | DAMAGING | 0 |
| rs34410516 | C/G | K8N | NP_000508 | N | DAMAGING | 0 |
| rs33914470 | C/G/T | K91N | NP_000508 | N | DAMAGING | 0 |
| rs33911106 | A/C/G/T | K91R | NP_000508 | R | DAMAGING | 0 |
| rs35993655 | C/T | L130P | NP_000549 | P | DAMAGING | 0 |
| rs34635364 | C/G/T | L137R | NP_000549 | R | DAMAGING | 0 |
| rs63749791 | C/G | L30V | NP_000508 | V | DAMAGING | 0.02 |
| rs35203445 | G/T | L35R | NP_000508 | R | DAMAGING | 0 |
| rs36030576 | G/T | L3R | NP_000508 | R | DAMAGING | 0 |
| rs34071856 | G/T | L81R | NP_000508 | R | DAMAGING | 0 |
| rs35548338 | G/T | L87R | NP_000508 | R | DAMAGING | 0 |
| rs17407508 | C/T | L92F | NP_000508 | F | DAMAGING | 0 |
| rs34684963 | C/T | L92P | NP_000508 | P | DAMAGING | 0 |
| rs34220980 | A/G | M1V | NP_000508 | V | DAMAGING | 0 |
| rs33969953 | A/C/G/T | M77K | NP_000508 | K | DAMAGING | 0 |
| rs33969953 | A/C/G/T | M77R | NP_000508 | R | DAMAGING | 0 |
| rs33969953 | A/C/G/T | M77T | NP_000508 | T | DAMAGING | 0 |
| rs28928885 | C/G | N10K | NP_000508 | K | DAMAGING | 0 |
| rs34823698 | A/C/G/T | N69D | NP_000508 | D | TOLERATED | 0.06 |
| rs33964623 | A/C/G | N79H | NP_000508 | H | TOLERATED | 0.08 |
| rs34440919 | C/G | N79K | NP_000508 | K | DAMAGING | 0.01 |
| rs41322954 | A/C | N98H | NP_000508 | H | DAMAGING | 0 |
| rs33910377 | C/G/T | P115L | NP_000508 | L | DAMAGING | 0 |
| rs33910377 | C/G/T | P115R | NP_000508 | R | DAMAGING | 0 |
| rs34472107 | C/G/T | P115S | NP_000508 | S | DAMAGING | 0 |
| rs63750566 | C/T | P120L | NP_000549 | L | DAMAGING | 0 |
| rs63750751 | C/T | P120S | NP_000549 | S | DAMAGING | 0 |
| rs35776155 | C/G/T | P38L | NP_000508 | L | DAMAGING | 0 |
| rs33978134 | C/G/T | P45L | NP_000508 | L | DAMAGING | 0 |
| rs33978134 | C/G/T | P45R | NP_000508 | R | DAMAGING | 0 |
| rs34019158 | A/C/G | P78H | NP_000508 | H | DAMAGING | 0 |
| rs33931314 | A/C/G/T | P96L | NP_000508 | L | DAMAGING | 0 |
| rs33931314 | A/C/G/T | P96Q | NP_000508 | Q | DAMAGING | 0 |
| rs33931314 | A/C/G/T | P96R | NP_000508 | R | DAMAGING | 0 |
| rs33984621 | A/C/G/T | P96S | NP_000508 | S | DAMAGING | 0 |
| rs35317336 | C/G | Q55E | NP_000508 | E | DAMAGING | 0 |
| rs36024711 | A/G | Q55R | NP_000508 | R | DAMAGING | 0 |
| rs33991910 | A/C/G/T | R142C | NP_000549 | C | DAMAGING | 0 |
| rs33991910 | A/C/G/T | R142G | NP_000549 | G | DAMAGING | 0 |
| rs33935328 | A/C/G/T | R142H | NP_000549 | H | DAMAGING | 0 |
| rs33935328 | A/C/G/T | R142L | NP_000549 | L | DAMAGING | 0 |
| rs33935328 | A/C/G/T | R142P | NP_000549 | P | DAMAGING | 0 |
| rs33991910 | A/C/G/T | R142S | NP_000549 | S | DAMAGING | 0 |
| rs33991779 | A/C/G/T | R93L | NP_000508 | L | DAMAGING | 0 |
| rs33991779 | A/C/G/T | R93P | NP_000508 | P | DAMAGING | 0 |
| rs33991779 | A/C/G/T | R93Q | NP_000508 | Q | DAMAGING | 0 |
| rs34868036 | C/T | R93W | NP_000508 | W | DAMAGING | 0 |
| rs34098449 | A/C | S103R | NP_000549 | R | DAMAGING | 0.01 |
| rs36008624 | C/T | S132F | NP_000549 | F | DAMAGING | 0 |
| rs35974739 | C/T | S132P | NP_000549 | P | DAMAGING | 0 |
| rs35082275 | A/G | S134N | NP_000549 | N | DAMAGING | 0 |
| rs55948437 | A/C | S134R | NP_000549 | R | DAMAGING | 0 |
| rs63749989 | C/G | S139C | NP_000549 | C | DAMAGING | 0 |
| rs34011123 | C/T | S139P | NP_000549 | P | DAMAGING | 0 |
| rs35850071 | C/T | S4F | NP_000508 | F | DAMAGING | 0 |
| rs34936612 | C/G | S82C | NP_000508 | C | DAMAGING | 0 |
| rs33926206 | A/C/G | S85G | NP_000508 | G | DAMAGING | 0 |
| rs33996798 | A/C/G | S85R | NP_000508 | R | DAMAGING | 0 |
| rs34890875 | A/T | T42S | NP_000508 | S | DAMAGING | 0 |
| rs63751008 | A/G | V122M | NP_000549 | M | DAMAGING | 0.03 |
| rs35166834 | G/T | V133G | NP_000549 | G | DAMAGING | 0 |
| rs35994191 | A/T | V136E | NP_000549 | E | DAMAGING | 0 |
| rs63751237 | A/G | V136M | NP_000549 | M | DAMAGING | 0 |
| rs34068598 | C/G | V56L | NP_000508 | L | DAMAGING | 0 |
| rs63750275 | A/G | V71M | NP_000508 | M | DAMAGING | 0 |
| rs34769782 | C/G/T | V94A | NP_000508 | A | DAMAGING | 0 |
| rs34769782 | C/G/T | V94G | NP_000508 | G | DAMAGING | 0 |
| rs33964317 | A/C/T | W15R | NP_000508 | R | DAMAGING | 0 |
| rs35723200 | C/T | Y141H | NP_000549 | H | DAMAGING | 0 |
| rs28928880 | A/G | Y25C | NP_000508 | C | DAMAGING | 0 |
| rs34743106 | C/T | Y25H | NP_000508 | H | DAMAGING | 0.04 |
