# Supplementary material for: In-Silico Computing of the Most Deleterious nsSNPs in HBA1 Gene
Source: PLoS One. 2016 Jan 29;11(1):e0147702. doi: 10.1371/journal.pone.0147702 (PMC4733110; doi:10.1371/journal.pone.0147702)
Supplement: S2 Table — (DOCX) [file pone.0147702.s005.docx]

**S2 Table.** SNAP2 score and the predicted effect of the variants on HBA1 protein.

| **Variant** | **Predicted Effect** | **Score** | **Expected Accuracy** |
| --- | --- | --- | --- |
| A111D | effect | 75 | 85% |
| A111T | effect | 42 | 71% |
| A112T | neutral | -73 | 87% |
| A116D | neutral | -46 | 72% |
| A121E | neutral | -43 | 72% |
| A124S | neutral | -30 | 61% |
| A131V | effect | 18 | 59% |
| A13D | effect | 13 | 59% |
| A14P | effect | 67 | 80% |
| A20E | neutral | -35 | 66% |
| A22D | effect | 59 | 75% |
| A22P | effect | 12 | 59% |
| A22V | neutral | -36 | 66% |
| A27V | effect | 7 | 53% |
| A54V | neutral | 0 | 53% |
| A64D | effect | 71 | 85% |
| A66V | effect | 58 | 75% |
| A6D | neutral | -54 | 78% |
| A6P | neutral | -11 | 57% |
| A72E | neutral | -32 | 66% |
| A72G | neutral | -51 | 78% |
| A72V | neutral | -21 | 61% |
| A80T | effect | 3 | 53% |
| A83D | neutral | -12 | 57% |
| A83T | neutral | -42 | 72% |
| A89S | effect | 33 | 66% |
| C105S | effect | 42 | 71% |
| D127E | effect | 33 | 66% |
| D127G | effect | 51 | 75% |
| D127H | effect | 43 | 71% |
| D127N | effect | 39 | 66% |
| D127V | effect | 44 | 71% |
| D127Y | effect | 53 | 75% |
| D48A | effect | 36 | 66% |
| D48G | effect | 44 | 71% |
| D48H | effect | 60 | 80% |
| D65G | neutral | -36 | 66% |
| D65H | effect | 19 | 59% |
| D65N | neutral | -17 | 57% |
| D65Y | effect | 25 | 63% |
| D75A | effect | 53 | 75% |
| D75G | effect | 58 | 75% |
| D75H | effect | 61 | 80% |
| D75N | effect | 57 | 75% |
| D76H | effect | 36 | 66% |
| D76N | neutral | -6 | 53% |
| D76V | effect | 60 | 80% |
| D76Y | effect | 60 | 80% |
| D7A | effect | 43 | 71% |
| D7G | effect | 55 | 75% |
| D7N | effect | 46 | 71% |
| D7V | effect | 60 | 80% |
| D7Y | effect | 67 | 80% |
| D86N | effect | 17 | 59% |
| D86Y | effect | 61 | 80% |
| D95E | effect | 36 | 66% |
| D95G | effect | 55 | 75% |
| D95H | effect | 41 | 71% |
| D95N | effect | 40 | 71% |
| D95Y | effect | 63 | 80% |
| E117A | neutral | -1 | 53% |
| E117K | effect | 39 | 66% |
| E117Q | effect | 4 | 53% |
| E24G | effect | 27 | 63% |
| E24V | neutral | -20 | 57% |
| E28D | effect | 18 | 59% |
| E28K | effect | 68 | 80% |
| E28V | effect | 49 | 71% |
| E31A | neutral | -47 | 72% |
| E31Q | neutral | -35 | 66% |
| E31V | neutral | -36 | 66% |
| F44V | effect | 75 | 85% |
| G16R | effect | 26 | 63% |
| G19D | effect | 24 | 63% |
| G19R | effect | 21 | 63% |
| G23D | effect | 23 | 63% |
| G52D | effect | 39 | 66% |
| G52R | effect | 31 | 66% |
| G52S | effect | 5 | 53% |
| G58D | effect | 34 | 66% |
| G58R | effect | 22 | 63% |
| G60A | effect | 41 | 71% |
| G60D | effect | 81 | 91% |
| G60V | effect | 67 | 80% |
| H104R | effect | 55 | 75% |
| H104Y | effect | 44 | 71% |
| H113D | effect | 63 | 80% |
| H113R | effect | 22 | 63% |
| H123Y | effect | 48 | 71% |
| H21D | effect | 30 | 66% |
| H21P | effect | 43 | 71% |
| H21R | effect | 10 | 59% |
| H46D | effect | 55 | 75% |
| H46P | effect | 56 | 75% |
| H46R | effect | 48 | 71% |
| H46Y | effect | 44 | 71% |
| H51L | effect | 25 | 63% |
| H51Q | effect | 14 | 59% |
| H51R | effect | 42 | 71% |
| H59Y | effect | 67 | 80% |
| H73D | effect | 50 | 75% |
| H73R | effect | 21 | 63% |
| H88P | effect | 92 | 95% |
| H88R | effect | 81 | 91% |
| H88Y | effect | 82 | 91% |
| H90L | effect | 26 | 63% |
| H90P | effect | 65 | 80% |
| H90Q | neutral | -9 | 53% |
| H90R | effect | 24 | 63% |
| H90Y | neutral | -33 | 66% |
| K100E | effect | 55 | 75% |
| K100N | effect | 38 | 66% |
| K128N | effect | 69 | 80% |
| K128T | effect | 64 | 80% |
| K12E | effect | 18 | 59% |
| K12Q | neutral | -28 | 61% |
| K140E | effect | 25 | 63% |
| K140T | effect | 7 | 53% |
| K41E | effect | 54 | 75% |
| K41M | effect | 21 | 63% |
| K41N | effect | 35 | 66% |
| K57E | effect | 57 | 75% |
| K57R | neutral | -14 | 57% |
| K57T | effect | 43 | 71% |
| K61E | effect | 35 | 66% |
| K62T | effect | 19 | 59% |
| K8N | effect | 48 | 71% |
| K91N | effect | 3 | 53% |
| K91R | neutral | -5 | 53% |
| L114R | effect | 67 | 80% |
| L130P | effect | 72 | 85% |
| L137R | effect | 76 | 85% |
| L30V | effect | 29 | 63% |
| L35R | effect | 31 | 66% |
| L3R | effect | 48 | 71% |
| L81R | effect | 80 | 91% |
| L87R | effect | 53 | 75% |
| L92F | effect | 41 | 71% |
| L92P | effect | 78 | 85% |
| K17E | effect | 46 | 71% |
| K17M | effect | 17 | 59% |
| K17T | effect | 28 | 63% |
| M1V | effect | 70 | 85% |
| M77K | effect | 77 | 85% |
| M77R | effect | 77 | 85% |
| M77T | effect | 60 | 80% |
| N10K | neutral | -2 | 53% |
| N69D | neutral | -48 | 72% |
| N69K | neutral | -46 | 72% |
| N79H | neutral | -62 | 82% |
| N79K | neutral | -22 | 61% |
| N98H | effect | 9 | 53% |
| P115L | effect | 50 | 75% |
| P115R | effect | 48 | 71% |
| P115S | effect | 16 | 59% |
| P120L | effect | 22 | 63% |
| P120S | effect | 1 | 53% |
| P38L | effect | 62 | 80% |
| P45L | effect | 39 | 66% |
| P45R | effect | 58 | 75% |
| P78H | neutral | -20 | 57% |
| P96L | effect | 65 | 80% |
| P96Q | effect | 62 | 80% |
| P96R | effect | 74 | 85% |
| P96S | effect | 50 | 75% |
| Q55E | effect | 36 | 66% |
| Q55R | effect | 42 | 71% |
| R142C | effect | 61 | 80% |
| R142G | effect | 80 | 91% |
| R142H | effect | 64 | 80% |
| R142L | effect | 65 | 80% |
| R142P | effect | 82 | 91% |
| R142S | effect | 72 | 85% |
| R93L | effect | 50 | 75% |
| R93P | effect | 71 | 85% |
| R93Q | effect | 47 | 71% |
| R93W | effect | 51 | 75% |
| S103R | effect | 70 | 85% |
| S132F | effect | 38 | 66% |
| S132P | effect | 49 | 71% |
| S134N | effect | 33 | 66% |
| S134R | effect | 67 | 80% |
| S139C | effect | 20 | 63% |
| S139P | effect | 42 | 71% |
| S4F | effect | 17 | 59% |
| S82C | neutral | -27 | 61% |
| S85G | effect | 27 | 63% |
| S85R | effect | 51 | 75% |
| T42S | effect | 7 | 53% |
| V122M | neutral | -41 | 72% |
| V133G | effect | 72 | 85% |
| V136E | effect | 44 | 71% |
| V136M | effect | 27 | 63% |
| V56L | neutral | -8 | 53% |
| V71M | effect | 47 | 71% |
| V94A | effect | 63 | 80% |
| V94G | effect | 82 | 91% |
| W15R | effect | 77 | 85% |
| Y141H | effect | 65 | 80% |
| Y25C | effect | 16 | 59% |
| Y25H | effect | 47 | 71% |
