# Supplementary material for: In-Silico Computing of the Most Deleterious nsSNPs in HBA1 Gene
Source: PLoS One. 2016 Jan 29;11(1):e0147702. doi: 10.1371/journal.pone.0147702 (PMC4733110; doi:10.1371/journal.pone.0147702)
Supplement: S3 Table — (DOCX) [file pone.0147702.s006.docx]

**S3 Table.** Wild and mutant residues of HBA1 protein and mCSM score.

| **Wild Residue** | **Residue Position** | **Mutant Residue** | **RSA (%)** | **score ΔΔG** |
| --- | --- | --- | --- | --- |
| A | 111 | D | 33.1 | -1.494 |
| A | 111 | T | 33.1 | -1.305 |
| A | 112 | T | 51.1 | -1.162 |
| A | 116 | D | 95.2 | -0.65 |
| A | 121 | E | 85 | -0.952 |
| A | 124 | S | 72.4 | -1.256 |
| A | 131 | V | 47 | -0.554 |
| A | 13 | D | 77.8 | -0.862 |
| A | 14 | P | 1.9 | 0.234 |
| A | 20 | E | 102.4 | -0.85 |
| A | 22 | D | 16.5 | -1.51 |
| A | 22 | P | 16.5 | -0.161 |
| A | 22 | V | 16.5 | -0.161 |
| A | 27 | V | 22.4 | -0.74 |
| A | 54 | V | 97.8 | -0.331 |
| A | 64 | D | 2.1 | -2.064 |
| A | 66 | V | 37.4 | -0.674 |
| A | 6 | D | 89 | -0.559 |
| A | 6 | P | 89 | -0.32 |
| A | 72 | E | 85.8 | -1.02 |
| A | 72 | G | 85.8 | -0.408 |
| A | 72 | V | 85.8 | -0.331 |
| A | 80 | T | 13.6 | -1.031 |
| A | 83 | D | 12.6 | -1.058 |
| A | 83 | T | 12.6 | -0.641 |
| A | 89 | S | 36 | -1.033 |
| C | 105 | S | 2.1 | -1.601 |
| D | 127 | E | 58.6 | -0.846 |
| D | 127 | G | 58.6 | -0.842 |
| D | 127 | H | 58.6 | -0.747 |
| D | 127 | N | 58.6 | -0.993 |
| D | 127 | V | 58.6 | 0.186 |
| D | 127 | Y | 58.6 | -0.084 |
| D | 48 | A | 61 | -0.518 |
| D | 48 | G | 61 | -0.721 |
| D | 48 | H | 61 | -0.655 |
| D | 65 | G | 64.3 | -0.47 |
| D | 65 | H | 64.3 | -0.788 |
| D | 65 | N | 64.3 | -0.216 |
| D | 65 | Y | 64.3 | -0.323 |
| D | 75 | A | 104.6 | -0.315 |
| D | 75 | G | 104.6 | -0.5 |
| D | 75 | H | 104.6 | -0.007 |
| D | 75 | N | 104.6 | -0.175 |
| D | 76 | H | 35.8 | -0.88 |
| D | 76 | N | 35.8 | -0.784 |
| D | 76 | V | 35.8 | 0.054 |
| D | 76 | Y | 35.8 | -0.379 |
| D | 7 | A | 21.2 | 0.408 |
| D | 7 | G | 21.2 | 0.2 |
| D | 7 | N | 21.2 | 0.132 |
| D | 7 | V | 21.2 | 0.756 |
| D | 7 | Y | 21.2 | -0.358 |
| D | 86 | N | 1.5 | -2.524 |
| D | 86 | Y | 1.5 | -2.983 |
| D | 95 | E | 32.6 | -0.596 |
| D | 95 | G | 32.6 | -0.352 |
| D | 95 | H | 32.6 | 0.269 |
| D | 95 | N | 32.6 | -1.035 |
| D | 95 | Y | 32.6 | 0.778 |
| E | 117 | A | 45.2 | -0.366 |
| E | 117 | K | 45.2 | -0.135 |
| E | 117 | Q | 45.2 | -0.613 |
| E | 24 | G | 76 | -1.142 |
| E | 24 | V | 76 | -0.232 |
| E | 28 | D | 32.8 | -1.325 |
| E | 28 | K | 32.8 | -0.71 |
| E | 28 | V | 32.8 | -0.046 |
| E | 31 | A | 31.8 | -0.86 |
| E | 31 | Q | 31.8 | -1.42 |
| E | 31 | V | 31.8 | -0.043 |
| F | 44 | V | 0 | -0.951 |
| G | 16 | R | 97.6 | -0.494 |
| G | 19 | D | 83.6 | -0.964 |
| G | 19 | R | 83.6 | -0.823 |
| G | 23 | D | 41.6 | -1.919 |
| G | 52 | D | 101.8 | -0.587 |
| G | 52 | R | 101.8 | -0.259 |
| G | 52 | S | 101.8 | -0.422 |
| G | 58 | D | 90.7 | -2.32 |
| G | 58 | R | 90.7 | -1.121 |
| G | 60 | A | 0 | -0.601 |
| G | 60 | D | 0 | -3.261 |
| G | 60 | V | 0 | -0.163 |
| H | 104 | R | 57 | -0.876 |
| H | 104 | Y | 57 | 0.93 |
| H | 113 | D | 39 | -1.023 |
| H | 113 | R | 39 | -1.03 |
| H | 123 | Y | 45.1 | 0.691 |
| H | 21 | D | 53 | -0.445 |
| H | 21 | P | 53 | 0.557 |
| H | 21 | R | 53 | -0.601 |
| H | 46 | D | 85.7 | -0.805 |
| H | 46 | P | 85.7 | 0.474 |
| H | 46 | R | 85.7 | -0.501 |
| H | 46 | Y | 85.7 | 0.835 |
| H | 51 | L | 87.8 | 0.49 |
| H | 51 | Q | 87.8 | -0.011 |
| H | 51 | R | 87.8 | -0.202 |
| H | 59 | Y | 5.2 | 0.238 |
| H | 73 | D | 39.1 | -0.273 |
| H | 73 | R | 39.1 | -0.478 |
| H | 88 | P | 6.6 | -1.367 |
| H | 88 | R | 6.6 | -0.956 |
| H | 88 | Y | 6.6 | -0.009 |
| H | 90 | L | 21.8 | -0.307 |
| H | 90 | P | 21.8 | -0.681 |
| H | 90 | Q | 21.8 | -1.102 |
| H | 90 | R | 21.8 | -0.754 |
| H | 90 | Y | 21.8 | 0.645 |
| K | 100 | E | 62.8 | 0.071 |
| K | 100 | N | 62.8 | -0.148 |
| K | 128 | N | 58.1 | 0.008 |
| K | 128 | T | 58.1 | -0.363 |
| K | 12 | E | 66.3 | 0.216 |
| K | 12 | Q | 66.3 | -0.043 |
| K | 140 | E | 62 | 0.16 |
| K | 140 | T | 62 | -0.515 |
| K | 41 | E | 51.7 | -0.305 |
| K | 41 | M | 51.7 | -0.207 |
| K | 41 | N | 51.7 | -1.017 |
| K | 57 | E | 86.2 | 0.397 |
| K | 57 | R | 86.2 | 0.072 |
| K | 57 | T | 86.2 | -0.051 |
| K | 61 | E | 66.4 | -0.241 |
| K | 62 | T | 66.8 | -0.84 |
| K | 8 | N | 51.2 | -0.219 |
| K | 91 | N | 23.8 | -1.576 |
| K | 91 | R | 23.8 | -0.649 |
| K | 17 | E | 56.7 | 0.099 |
| K | 17 | M | 56.7 | 0.16 |
| K | 17 | T | 56.7 | -0.308 |
| L | 114 | R | 10.2 | -1.503 |
| L | 130 | P | 2.4 | -1.492 |
| L | 137 | R | 1.2 | -1.067 |
| L | 30 | V | 0.3 | -1.716 |
| L | 35 | R | 93.4 | 0.082 |
| L | 3 | R | 91.7 | 0.073 |
| L | 81 | R | 79.4 | -0.084 |
| L | 87 | R | 2 | -1.792 |
| L | 92 | F | 92.8 | -0.912 |
| L | 92 | P | 92.8 | -0.734 |
| M | 77 | K | 12.8 | -1.256 |
| M | 77 | R | 12.8 | -1.054 |
| M | 77 | T | 12.8 | -1.604 |
| N | 10 | K | 30.2 | -0.113 |
| N | 69 | D | 64.4 | -0.535 |
| N | 69 | K | 64.4 | -0.11 |
| N | 79 | H | 79.7 | -0.736 |
| N | 79 | K | 79.7 | -0.099 |
| N | 98 | H | 1.6 | -1.481 |
| P | 115 | L | 70.2 | -0.342 |
| P | 115 | R | 70.2 | -0.095 |
| P | 115 | S | 70.2 | -0.778 |
| P | 120 | L | 93.7 | -0.277 |
| P | 120 | S | 93.7 | -0.436 |
| P | 38 | L | 59.8 | -0.398 |
| P | 45 | L | 66.6 | -0.321 |
| P | 45 | R | 66.6 | -0.179 |
| P | 78 | H | 49.5 | -1.002 |
| P | 96 | L | 48.3 | -0.474 |
| P | 96 | Q | 48.3 | -1.13 |
| P | 96 | R | 48.3 | -0.714 |
| P | 96 | S | 48.3 | -1.531 |
| Q | 55 | E | 53.3 | -0.778 |
| Q | 55 | R | 53.3 | -0.545 |
| R | 93 | L | 84.9 | 0.76 |
| R | 93 | P | 84.9 | 0.536 |
| R | 93 | Q | 84.9 | 0.198 |
| R | 93 | W | 84.9 | 0.139 |
| S | 103 | R | 13.9 | -0.86 |
| S | 132 | F | 38 | -1.244 |
| S | 132 | P | 38 | -0.466 |
| S | 134 | N | 8 | -1.01 |
| S | 134 | R | 8 | -0.581 |
| S | 139 | C | 68.6 | -0.291 |
| S | 139 | P | 68.6 | -0.251 |
| S | 4 | F | 49 | -0.855 |
| S | 82 | C | 16.5 | -0.399 |
| S | 85 | G | 0 | -1.543 |
| S | 85 | R | 0 | -0.912 |
| T | 42 | S | 85.2 | -0.691 |
| V | 122 | M | 20.7 | -0.841 |
| V | 133 | G | 4.9 | -2.465 |
| V | 136 | E | 21.9 | -2.63 |
| V | 136 | M | 21.9 | -0.977 |
| V | 56 | L | 7.3 | -0.568 |
| V | 71 | M | 15.7 | -0.352 |
| V | 94 | A | 0 | -1.498 |
| V | 94 | G | 0 | -1.966 |
| W | 15 | R | 8.1 | -2.276 |
| Y | 25 | C | 8.1 | -1.786 |
| Y | 25 | H | 8.1 | -1.963 |

RSA: Relative Solvent Accessible Area
