# Supplementary material for: In-Silico Computing of the Most Deleterious nsSNPs in HBA1 Gene
Source: PLoS One. 2016 Jan 29;11(1):e0147702. doi: 10.1371/journal.pone.0147702 (PMC4733110; doi:10.1371/journal.pone.0147702)
Supplement: S4 Table — (DOCX) [file pone.0147702.s007.docx]

**S4 Table.** Prediction and probability of variants of HBA1 protein using SNPs&GO.

| **Mutation** | **Prediction** | **RI** | **Probability** |
| --- | --- | --- | --- |
| A6D | Neutral | 6 | 0.213 |
| A6P | Neutral | 5 | 0.254 |
| D7A | Neutral | 7 | 0.161 |
| D7G | Neutral | 5 | 0.256 |
| D7N | Neutral | 7 | 0.164 |
| D7V | Neutral | 4 | 0.288 |
| D7Y | Neutral | 5 | 0.258 |
| A13D | Neutral | 2 | 0.393 |
| A14P | Disease | 4 | 0.697 |
| G16R | Neutral | 2 | 0.401 |
| G19D | Neutral | 5 | 0.269 |
| G19R | Neutral | 4 | 0.305 |
| A20E | Neutral | 5 | 0.271 |
| H21D | Neutral | 1 | 0.448 |
| A22D | Neutral | 0 | 0.486 |
| A22P | Disease | 1 | 0.554 |
| A22V | Neutral | 7 | 0.126 |
| G23D | Neutral | 7 | 0.15 |
| E24G | Neutral | 2 | 0.383 |
| E24V | Disease | 1 | 0.567 |
| A27V | Neutral | 3 | 0.335 |
| E28D | Disease | 1 | 0.548 |
| E28K | Disease | 6 | 0.783 |
| E28V | Disease | 7 | 0.848 |
| E31A | Neutral | 8 | 0.084 |
| E31Q | Neutral | 8 | 0.091 |
| E31V | Neutral | 4 | 0.315 |
| F44V | Disease | 8 | 0.886 |
| D48A | Neutral | 2 | 0.415 |
| D48G | Neutral | 2 | 0.41 |
| D48H | Disease | 1 | 0.544 |
| G52D | Disease | 6 | 0.812 |
| G52R | Disease | 6 | 0.795 |
| G52S | Disease | 2 | 0.61 |
| A54V | Neutral | 3 | 0.344 |
| G58D | Disease | 1 | 0.549 |
| G58R | Neutral | 1 | 0.463 |
| G60A | Disease | 1 | 0.55 |
| G60D | Disease | 6 | 0.788 |
| G60V | Disease | 6 | 0.815 |
| A64D | Disease | 4 | 0.679 |
| D65G | Neutral | 8 | 0.116 |
| D65H | Neutral | 7 | 0.147 |
| D65N | Neutral | 8 | 0.121 |
| D65Y | Neutral | 1 | 0.463 |
| A66V | Disease | 5 | 0.756 |
| A72E | Neutral | 5 | 0.262 |
| A72G | Neutral | 8 | 0.112 |
| A72V | Neutral | 6 | 0.219 |
| D75A | Disease | 2 | 0.581 |
| D75G | Disease | 3 | 0.643 |
| D75H | Disease | 3 | 0.633 |
| D75N | Disease | 1 | 0.535 |
| D76H | Neutral | 2 | 0.413 |
| D76N | Neutral | 7 | 0.139 |
| D76V | Disease | 2 | 0.62 |
| D76Y | Disease | 1 | 0.572 |
| A80T | Neutral | 7 | 0.144 |
| A83D | Neutral | 6 | 0.201 |
| A83T | Neutral | 9 | 0.059 |
| D86N | Neutral | 7 | 0.13 |
| D86Y | Disease | 0 | 0.504 |
| A89S | Neutral | 3 | 0.343 |
| D95E | Neutral | 0 | 0.495 |
| D95G | Disease | 3 | 0.634 |
| D95H | Disease | 3 | 0.662 |
| D95N | Disease | 1 | 0.555 |
| D95Y | Disease | 5 | 0.77 |
| H104R | Neutral | 5 | 0.265 |
| H104Y | Neutral | 3 | 0.34 |
| C105S | Disease | 0 | 0.518 |
| A111D | Disease | 6 | 0.814 |
| A111T | Disease | 4 | 0.685 |
| A112T | Neutral | 9 | 0.07 |
| H113D | Neutral | 2 | 0.389 |
| H113R | Neutral | 2 | 0.394 |
| A116D | Neutral | 4 | 0.29 |
| E117A | Neutral | 9 | 0.057 |
| E117K | Neutral | 4 | 0.277 |
| E117Q | Neutral | 7 | 0.172 |
| A121E | Neutral | 8 | 0.089 |
| H123Y | Disease | 1 | 0.547 |
| A124S | Neutral | 2 | 0.422 |
| D127E | Neutral | 3 | 0.363 |
| D127G | Disease | 3 | 0.643 |
| D127H | Disease | 2 | 0.62 |
| D127N | Neutral | 1 | 0.464 |
| D127V | Disease | 5 | 0.757 |
| D127Y | Disease | 5 | 0.763 |
| A131V | Neutral | 6 | 0.223 |
| M1V | Neutral | 8 | 0.125 |
| L3R | Neutral | 4 | 0.279 |
| K8N | Neutral | 4 | 0.295 |
| N10K | Neutral | 5 | 0.269 |
| K12E | Neutral | 0 | 0.488 |
| K12Q | Neutral | 7 | 0.163 |
| K17E | Disease | 7 | 0.857 |
| K17M | Disease | 4 | 0.718 |
| K17T | Disease | 6 | 0.824 |
| H21P | Disease | 7 | 0.839 |
| H21R | Neutral | 1 | 0.43 |
| L30V | Neutral | 5 | 0.263 |
| L35R | Neutral | 3 | 0.366 |
| P38L | Disease | 7 | 0.865 |
| K41E | Disease | 2 | 0.621 |
| K41M | Disease | 1 | 0.562 |
| K41N | Disease | 2 | 0.605 |
| P45L | Neutral | 2 | 0.39 |
| P45R | Neutral | 1 | 0.471 |
| H46D | Disease | 3 | 0.664 |
| H46P | Disease | 7 | 0.828 |
| H46R | Disease | 4 | 0.694 |
| H46Y | Disease | 2 | 0.593 |
| H51L | Neutral | 2 | 0.379 |
| H51Q | Neutral | 6 | 0.196 |
| H51R | Neutral | 4 | 0.28 |
| Q55E | Neutral | 4 | 0.311 |
| Q55R | Neutral | 4 | 0.315 |
| K57E | Disease | 1 | 0.551 |
| K57R | Neutral | 8 | 0.077 |
| K57T | Neutral | 2 | 0.398 |
| H59Y | Disease | 7 | 0.826 |
| K61E | Disease | 0 | 0.501 |
| K62T | Disease | 3 | 0.649 |
| N69D | Neutral | 8 | 0.095 |
| N69K | Neutral | 8 | 0.088 |
| H73D | Disease | 0 | 0.504 |
| H73R | Disease | 1 | 0.553 |
| M77K | Disease | 2 | 0.596 |
| M77R | Disease | 4 | 0.676 |
| M77T | Neutral | 3 | 0.328 |
| P78H | Neutral | 4 | 0.31 |
| N79H | Neutral | 8 | 0.099 |
| N79K | Neutral | 8 | 0.119 |
| L81R | Disease | 6 | 0.824 |
| L87R | Disease | 6 | 0.779 |
| H88P | Disease | 7 | 0.829 |
| H88R | Disease | 6 | 0.78 |
| H88Y | Disease | 4 | 0.721 |
| H90L | Neutral | 3 | 0.346 |
| H90P | Disease | 5 | 0.775 |
| H90Q | Neutral | 3 | 0.363 |
| H90R | Neutral | 1 | 0.427 |
| H90Y | Neutral | 9 | 0.049 |
| K91N | Neutral | 5 | 0.259 |
| K91R | Neutral | 6 | 0.194 |
| L92F | Disease | 5 | 0.742 |
| L92P | Disease | 7 | 0.848 |
| R93L | Disease | 2 | 0.611 |
| R93P | Disease | 6 | 0.783 |
| R93Q | Neutral | 1 | 0.469 |
| R93W | Disease | 2 | 0.605 |
| P96L | Disease | 7 | 0.866 |
| P96Q | Disease | 8 | 0.911 |
| P96R | Disease | 8 | 0.881 |
| P96S | Disease | 7 | 0.867 |
| N98H | Disease | 3 | 0.651 |
| K100E | Disease | 4 | 0.676 |
| K100N | Disease | 1 | 0.56 |
| S103R | Disease | 2 | 0.619 |
| L114R | Disease | 1 | 0.53 |
| P115L | Disease | 4 | 0.705 |
| P115R | Disease | 5 | 0.735 |
| P115S | Neutral | 2 | 0.401 |
| P120L | Disease | 5 | 0.744 |
| P120S | Disease | 3 | 0.64 |
| K128N | Disease | 5 | 0.773 |
| K128T | Disease | 6 | 0.821 |
| L130P | Disease | 6 | 0.785 |
| S132F | Neutral | 2 | 0.411 |
| S132P | Disease | 1 | 0.575 |
| S134N | Neutral | 3 | 0.348 |
| L137R | Disease | 6 | 0.795 |
| K140E | Neutral | 1 | 0.431 |
| K140T | Neutral | 4 | 0.307 |
| R142C | Disease | 3 | 0.63 |
| R142G | Disease | 1 | 0.559 |
| R142H | Disease | 2 | 0.591 |
| R142L | Disease | 4 | 0.692 |
| R142P | Disease | 3 | 0.644 |
| R142S | Disease | 4 | 0.677 |
| S4F | Neutral | 8 | 0.118 |
| W15R | Disease | 7 | 0.853 |
| Y25C | Disease | 2 | 0.61 |
| Y25H | Neutral | 5 | 0.234 |
| T42S | Neutral | 2 | 0.394 |
| V56L | Neutral | 5 | 0.246 |
| V71M | Neutral | 3 | 0.338 |
| S82C | Neutral | 3 | 0.356 |
| S85G | Neutral | 2 | 0.394 |
| S85R | Disease | 4 | 0.698 |
| V94A | Disease | 1 | 0.536 |
| V94G | Disease | 4 | 0.719 |
| V122M | Neutral | 8 | 0.109 |
| V133G | Disease | 4 | 0.703 |
| S134R | Disease | 1 | 0.542 |
| V136E | Disease | 6 | 0.784 |
| V136M | Neutral | 3 | 0.333 |
| S139C | Neutral | 6 | 0.176 |
| S139P | Neutral | 2 | 0.386 |
| Y141H | Neutral | 3 | 0.362 |

RI: Reliability Index; Probability: Disease probability (if >0.5 mutation is predicted Disease)
